# Combined effect of living alone and osteoporosis status on the prevalence of depression in Chinese community-dwelling older population: a cross-sectional study

**DOI:** 10.3389/fpubh.2026.1729283

**Published:** 2026-01-15

**Authors:** Pingping Cai, Siqin Gaowa, Cheng Lin, Peipei Han, Xiaoyu Chen, Jinwen Zhang, Cheng Chen, Qianhao Wu, Jingjie Miao, Shumei Zhang, Lihua Li, Talin SarNa, Qi Guo

**Affiliations:** 1 School of Health, Fujian Medical University, Fuzhou, Fujian, China; 2 College of Rehabilitation Sciences, Shanghai University of Medicine and Health Sciences, Shanghai, China; 3 Department of Cardiovascular Medicine, Inner Mongolia People’s Hospital, Inner Mongolia, China; 4 Department of Rehabilitation Medicine, Shanghai University of Medicine and Health Sciences Affiliated Zhoupu Hospital, Shanghai, China; 5 Graduate School, Shanghai University of Traditional Chinese Medicine, Shanghai, China

**Keywords:** depression, older population, living alone, osteoporosis, social contact

## Abstract

**Background:**

Given the increasing burden of the “silent” depressive symptoms and the significant comorbidity of osteoporosis in the population living alone, this study aimed to investigate the separate and combined impacts of living alone and osteoporosis on the prevalence of depression in the older Chinese population.

**Methods:**

A total of 4,240 Chinese suburban-dwelling older individuals (mean age: 71.75 ± 5.88 years; 58.0% women) aged ≥60 years were recruited. Living arrangements were assessed by a questionnaire. Participants with a T score less than or equal to −2.5 were identified as osteoporosis. Participants were categorized into four groups based on their living status and osteoporosis prevalence: living with others and non-osteoporosis; living with others and osteoporosis; living alone and non-osteoporosis and living alone and osteoporosis groups. Depression was assessed by the Chinese version of Geriatric Depressive symptoms Scale (GDS).

**Results:**

515 (12.1%) were measured to have depressive symptoms (141 males and 374 females). In males, a significant association with depression only existed in the combined group of living alone and osteoporosis (OR = 3.61, 95%CI = 1.78–7.30). However, living alone with or without osteoporosis showed a significantly higher prevalence of depression in females (OR = 2.11, 95%CI = 1.34–3.31; OR = 2.21, 95%CI = 1.44–3.39, respectively).

**Conclusion:**

Osteoporosis by itself had no significant association with depression. However, the combination of living alone and osteoporosis was significantly associated with a higher prevalence of depression, especially in males. This study highlights the critical need for early identification and appropriate intervention for osteoporosis among older individuals living alone.

## 1 Introduction

As one of the most prevalent mental disorders among the older adults, depression significantly impairs their quality of life and psychological well-being (1). The prevalence of depression among older adults is 22.7%, with rates of 19.4% in males and 24.2% in females (2). However, depression remains frequently underrecognized and undertreated, partly due to the reluctance or difficulty many older adults experience in articulating emotional distress (3, 4). Consequently, low rates of depression-specific clinical care and treatment have been reported (5). Individuals living alone may be particularly vulnerable, as they often experience lower levels of social support (6), a factor consistently linked to higher depression risk (7–9). Given that mental health concerns in older people living alone are especially prone to be neglected, they warrant focused clinical and public health attention (4).

With the advancement of global aging, both the number and proportion of older adults living alone are rising (6). Females are more likely to experience spousal loss and subsequently live alone due to sex-based differences in life expectancy (10). Research have shown that living alone is associated with a range of adverse health outcomes, including cardiovascular diseases, frailty, reduced bone mineral density, and increased mortality rates (11–13). Consequently, the health status of this growing demographic constitutes a significant public health concern. Therefore, it is imperative to examine how additional factors affect depression for people with different living arrangements.

Osteoporosis was the most prevalent comorbid condition among older people living alone in the community (14). It is characterized as a chronic systemic bone disease in older adults, especially women (15, 16). Evidence indicates that osteoporosis serves as an independent risk factor for depression (15, 17). The two conditions shared multiple risk factors and similar physiopathologic basis, including interleukin-6 (IL-6), C-reactive protein (CRP), cortisol (18). Furthermore, while physical activity confers a protective effect against depression (19), individuals with osteoporosis often exhibit reduced levels of exercise (20), potentially establishing a self-reinforcing cycle. But the reported magnitude and direction of the association between osteoporosis and depression remain inconsistent across studies (15, 21).

Although some studies have focused on the separate effects of living alone and osteoporosis on depression in older adults, the results remain controversial (7, 9, 15, 21–23). Furthermore, their combined effect on depression remains unclear. Given the potential gender differences in these relationships (3, 10, 24), we aimed to explore the independent and joint associations of living alone and osteoporosis with depression among males and females separately.

## 2 Methods

### 2.1 Study participants

**Figure 1:**
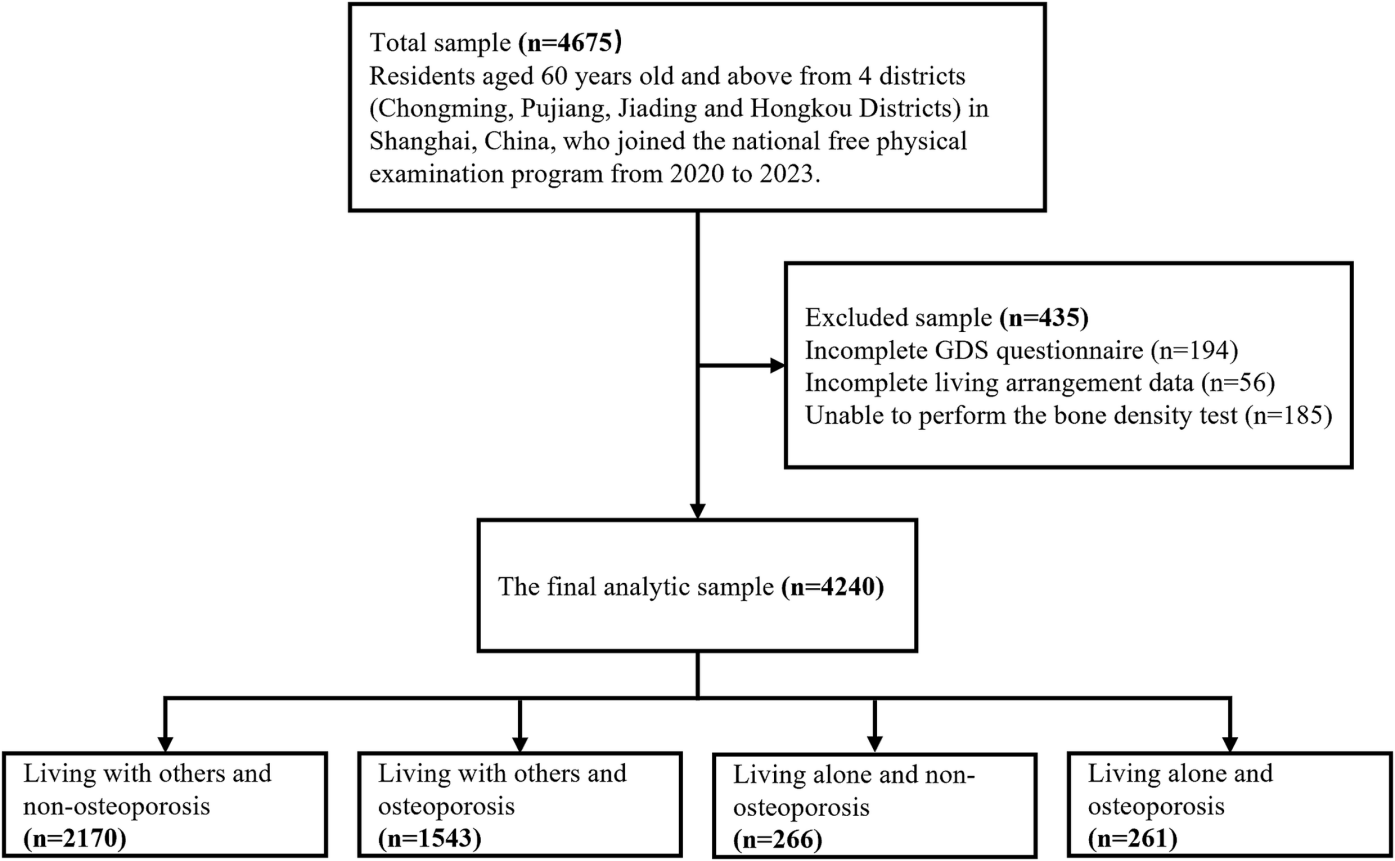
Flow chart of participant selection.

Our study population comprised 4,675 suburban-dwelling older individuals (aged 60 years old and above) from the Chongming, Pujiang, Jiading and Hongkou Districts of Shanghai, China who were participated in China’s national free physical examination program between 2020 and 2023. The inclusion criteria for participants were age ≥60 years, willingness to participate in this study, and completion of the relevant information. Exclusion criteria were as follows: (1) those with incomplete depression data; (2) those with incomplete living arrangements information; (3) those unable to perform the bone density test. The final analytic data consisted of 4,240 participants (Figure 1).

This study was approved by the ethical committee of the Shanghai University of Medicine and Health Sciences, and all participants gave full, informed written consent to take part in the study.

### 2.2 Assessment of living alone

Living arrangements were assessed by a questionnaire item: “How many people are in your household, except yourself?.” Participants were defined as living alone if they are the only member in the household. Those who reported any co-resident were classified as living with others (25, 26).

### 2.3 Assessment of osteoporosis

The bone mineral density (BMD) was measured at the distal one-third radius of the non-stressed forearm by the portable dual-energy X-ray absorptiometry (EXA-3000; OsteoSys, Co., Ltd., 901-914, 9F, Jnk Digitaltower, 111 Digital-ro 26, Guro-gu, Seoul 152-848, Republic of Korea), which was calibrated by trained technicians every day. Based on World Health Organization (WHO) criteria (27), a T score ≤ − 2.5 was considered to indicate osteoporosis.

### 2.4 Assessment of depressive symptoms

Depressive symptoms were diagnosed by an interview using the Chinese version of Geriatric Depressive symptoms Scale (GDS). GDS is a standardized self-reported questionnaire comprising 30 dichotomous questions, with a total score ranging 0–30. And greater values indicate increased severity. Participants with a GDS score 11 or above were defined as those with depressive symptoms (28).

### 2.5 Covariates

In this study, we controlled for the following variables as covariates: sociodemographic characteristics, behavioral characteristics, condition of chronic diseases.

Demographic characteristics, including age, gender, social contact, monthly income, and education level were assessed. Behavioral characteristics included smoking and drinking habits. Current smoking habits were grouped into 2 categories: current smoker, non-smoker. Current drinking habits were grouped into 2 categories: current drinker, non-drinker. Social contact was assessed with the following question: “Do you regularly interact with family or friends in daily life?.” Participants were divided into 3 categories: none, irregular, and regular. Education level was grouped into 2 categories: illiterate and non-illiterate.

We used the short form of the International Physical Activity Questionnaire (IPAQ) to assess physical activity (29). The nutrition status was measured using the Mini Nutritional Assessment (MNA) (30). The history of medical conditions such as diabetes, hypertension, hyperlipidemia and stroke was established according to his or her response (yes or no) to questions, the physician’s diagnosis and medication or treatment.

### 2.6 Statistical analysis

Participants were divided into four groups to determine combined conditions of living alone and osteoporosis status. Four groups, respectively, are living with others and non-osteoporosis; living with others and osteoporosis; living alone and non-osteoporosis and living alone and osteoporosis groups.

Continuous variables were presented as the mean ± standard deviation (SD), whereas data with a non-normal distribution were expressed as the median (25–75% interquartile range). Categorical variables were summarized as an absolute number and proportion (%). Comparisons among four different groups were examined using analysis of variance, Kruskal-Wallis rank tests and Chi-square tests. Furthermore, a binary logistic regression analyses were performed to examine the association of living alone or osteoporosis with depressive symptoms in older adults. Analyses were conducted in crude (Model 1 and Model 2) and adjusted (Model 3, Model 4, and Model 5) models. All statistical analyses were performed with SPSS V25 and *p* values less than 0.05 were considered statistically significant.

## 3 Results

### 3.1 Characteristics of participants among four groups classified by living alone and osteoporosis status

Of the 4,240 (mean age, 71.75 ± 5.88 years; 58.0% women) in the final analysis, 515 (12.1%) were assessed to have depressive symptoms (7.9% in males and 15.2% in females). Table 1 displays the characteristics between participants with and without depression. Depressed individuals were older, females, more likely to live alone, and had lower BMI, lower MNA score and a higher prevalence of osteoporosis. Table 2 categorizes participants by living alone and osteoporosis status. Compared to the other three groups, participants in the living alone and osteoporosis group were older, predominantly female, had lower IPAQ scores, more often illiterate, and exhibited the highest frequency of depressive symptoms.

**Table 1:** Characteristics of the study population according to the depression rate.

Characteristic	Non-depressed group (*n* = 3,725)	Depressed group (*n* = 515)	*p*-value
Age, year	71.58 ± 5.79	73.02 ± 6.40	<0.001
Gender, *n* (%)			<0.001
Male	1,641 (44.1)	141 (27.4)	
Female	2084 (55.9)	374 (72.6)	
BMI, kg/m^2^	23.99 ± 3.22	23.64 ± 3.35	0.019
IPAQ, Mets/week	4053.0 (1533.0, 7413.0)	2653.0 (720.0, 5040.0)	<0.001
MNA, score	26.33 ± 2.24	24.61 ± 2.91	<0.001
Living alone, *n* (%)	408 (11.0)	119 (23.1)	<0.001
Osteoporosis, *n* (%)	1,531 (41.1)	273 (53.0)	<0.001
Social contact, *n* (%)			<0.001
None	794 (21.7)	166 (32.4)	
Irregular	1,310 (35.8)	180 (35.1)	
Regular	1,560 (42.7)	167 (32.5)	
Monthly income (RMB), *n* (%)			<0.001
<3,000	1,046 (28.2)	206 (40.3)	
3,000–5,000	806 (21.8)	119 (23.2)	
>5,000	1851 (50.0)	187 (36.5)	
Education level, *n* (%)			<0.001
Illiterate	260 (7.0)	72 (14.0)	
Non-illiterate	3,449 (93.0)	442 (86.0)	
Current smoking, *n* (%)	539 (14.5)	40 (7.8)	<0.001
Current drinking, *n* (%)	856 (23.1)	66 (12.9)	<0.001
Disease history, *n* (%)			
Diabetes	681 (18.3)	109 (21.2)	0.115
Hypertension	2,245 (60.3)	321 (62.3)	0.370
Hyperlipidemia	903 (24.2)	153 (29.7)	0.007
Stroke	487 (13.1)	140 (27.2)	<0.001

BMI, body mass index; IPAQ, International Physical Activity Questionnaire; Mets/week, metabolic equivalent task minutes per week; MNA, mini-nutritional assessment.

**Table 2:** Characteristics of the study population according to categories of living alone and prevalence of osteoporosis.

Characteristic	Living with others and non-osteoporosis (*n* = 2,170)	Living with others and osteoporosis (*n* = 1,543)	Living alone and non-osteoporosis (*n* = 266)	Living alone and osteoporosis (*n* = 261)	*p*-value
Age, year	70.3 ± 5.2	73.0 ± 6.0^a^	71.8 ± 6.0^a^^,^^b^	76.1 ± 6.6^a^^,^^b^^,^^c^	<0.001
Gender, *n* (%)					<0.001
Male	1,077 (49.6)	555 (36.0)^a^	92 (34.6)^a^	58 (22.2)^a^^,^^b^^,^^c^	
Female	1,093 (50.4)	988 (64.0)^a^	174 (65.4)^a^	203 (77.8)^a^^,^^b^^,^^c^	
BMI, kg/m^2^	24.4 ± 3.3	23.4 ± 3.1^a^	24.3 ± 3.3^b^	23.1 ± 3.1^a^^,^^c^	<0.001
IPAQ, Mets/week	4399.5 (1533.0,7759.5)	3759.0 (1386.0,7092.0)^a^	3966.0 (1413.0,7112.3)	2772.0 (1053.0,5292.0)^a^^,^^b^^,^^c^	<0.001
MNA, score	26.4 ± 2.3	25.8 ± 2.4^a^	26.3 ± 2.3^b^	25.4 ± 2.6^a^^,^^c^	<0.001
Social contact, *n* (%)					<0.001
None	439 (20.5)	361 (23.8)	85 (32.4)^a^^,^^b^	75 (28.8)^a^	
Irregular	747 (35.0)	557 (36.7)	85 (32.4)	101 (38.8)	
Regular	952 (44.5)	599 (39.5)^a^	92 (35.2)^a^	84 (32.4)^a^	
Monthly income (RMB), *n* (%)					<0.001
<3,000	546 (25.2)	426 (27.9)	147 (55.5)^a^^,^^b^	133 (51.0)^a^^,^^b^	
3,000–5,000	460 (21.3)	304 (19.9)	78 (29.4)^a^^,^^b^	83 (31.8)^a^^,^^b^	
>5,000	1,157 (53.5)	796 (52.2)	40 (15.1)^a^^,^^b^	45 (17.2)^a^^,^^b^	
Education level, *n* (%)					<0.001
Illiterate	121 (5.6)	143 (9.3)^a^	20 (7.5)	48 (18.5)^a^^,^^b^^,^^c^	
Non-illiterate	2038 (94.4)	1,396 (90.7)^a^	245 (92.5)	212 (81.5)^a^^,^^b^^,^^c^	
Current smoking, *n* (%)	343 (15.8)	174 (11.3)^a^	38 (14.3)	24 (9.2)^a^	<0.001
Current drinking, *n* (%)	552 (25.5)	279 (18.1)^a^	50 (18.8)	41 (15.7)^a^	<0.001
Depression, *n* (%)	195 (9.0)	201 (13.0)^a^	47 (17.7)^a^	72 (27.6)^a^^,^^b^^,^^c^	<0.001
Disease history, *n* (%)					
Diabetes	405 (18.7)	281 (18.2)	58 (21.8)	46 (17.6)	0.547
Hypertension	1,333 (61.4)	908 (58.8)	162 (60.9)	163 (62.5)	0.394
Hyperlipidemia	545 (25.1)	370 (24.0)	68 (25.6)	73 (28.0)	0.546
Stroke	292 (13.5)	239 (15.5)	53 (19.9)^a^	43 (16.5)	0.020

a *p* < 0.05 versus the “living with others and non-osteoporosis” group.

b *p* < 0.05 versus the “living with others and osteoporosis” group.

c *p* < 0.05 versus the “living alone and non-osteoporosis” group.

BMI, body mass index; IPAQ, International Physical Activity Questionnaire; Mets/week, metabolic equivalent task minutes per week; MNA, mini-nutritional assessment.

**Figure 2:**
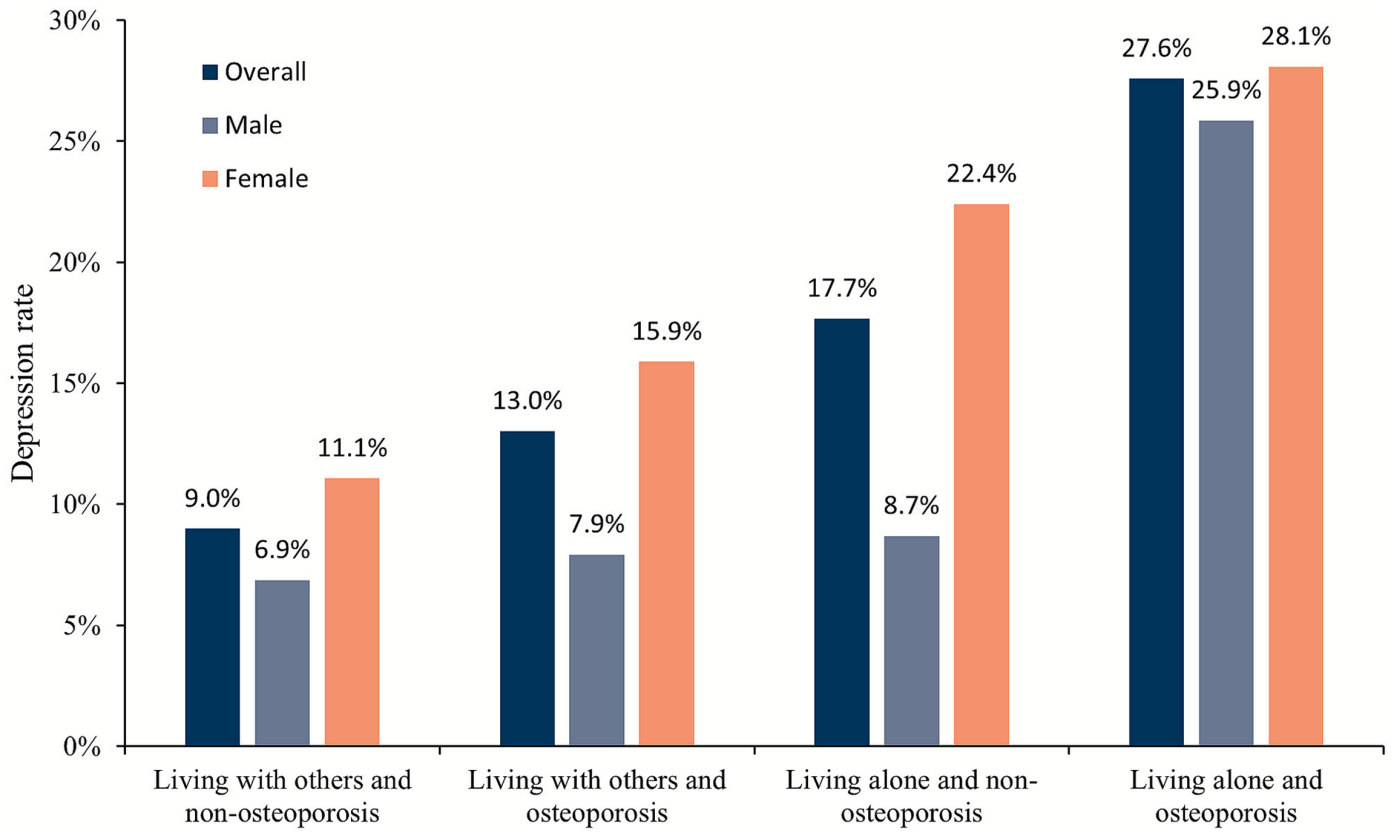
Depression rate based on categories of living alone and osteoporosis status among the study population in overall, male, and female participants.

As shown in Figure 2, the prevalence of depression was highest in the living alone and osteoporosis group and lowest in the living with others and non-osteoporosis group; this pattern was consistent across both sexes.

### 3.2 The association between living alone, osteoporosis status and depressive symptoms

The analyses were performed separately for males and females due to the known gender differences in depression (20). Tables 3, 4, respectively, showed the logistic regression analyses for living alone and osteoporosis with depression by gender. We found that living alone was significantly associated with depressive symptoms for the males and females in model 1 (all *p* < 0.05). Model 2 in females showed that osteoporosis was significantly associated with depressive symptoms (*p* < 0.001). After adjusting for potential confounders (age, BMI, IPAQ, MNA score, social contact, monthly income, education level, current smoking, current drinking, diabetes, hypertension, stroke), we observed that living alone (OR = 2.03, 95%CI = 1.20–3.41 in males; OR = 1.86, 95%CI = 1.37–2.54 in females) was significantly correlated with depressive symptoms. However, we did not find any statistically significant differences between osteoporosis and depressive symptoms both for males and females in model 4. Compared with the living with others and non-osteoporosis group, we found that only the living alone and osteoporosis group showed statistically significant association after adjusting for potential confounders in males (*p* < 0.001). However, in females, participants living alone, regardless of osteoporosis status, had greater adjusted risks of depression (all *p* < 0.05).

**Table 3:** Logistic regression analyses for living alone and osteoporosis with depression in the male.

Characteristic	Model 1	Model 2	Model 3	Model 4	Model 5
	Crude model OR (95% CI)	Crude model OR (95% CI)	Adjusted model OR (95% CI)	Adjusted model OR (95% CI)	Adjusted model OR (95% CI)
Living arrangement					
Living with others	Reference		Reference		
Living alone	2.32 (1.44, 3.76)		2.03 (1.20, 3.41)		
Osteoporosis					
No		Reference		Reference	
Yes		1.41 (0.99, 2.00)		1.17 (0.79, 1.72)	
Living arrangement and osteoporosis					
Living with others and non-osteoporosis					Reference
Living with others and osteoporosis					0.97 (0.63, 1.48)
Living alone and non-osteoporosis					1.11 (0.50, 2.47)
Living alone and osteoporosis					3.61 (1.78, 7.30)
Age			1.01 (0.98, 1.04)	1.01 (0.98, 1.04)	1.01 (0.97, 1.04)
BMI			1.08 (1.01, 1.16)	1.08 (1.01,1.16)	1.08 (1.01, 1.15)
IPAQ			0.92 (0.87, 0.96)	0.92 (0.87, 0.97)	0.92 (0.87, 0.97)
MNA score			0.75 (0.69, 0.82)	0.76 (0.69, 0.82)	0.76 (0.70, 0.83)
Social contact					
None			Reference	Reference	Reference
Irregular			0.93 (0.58, 1.47)	0.89 (0.56, 1.42)	0.92 (0.58, 1.47)
Regular			0.97 (0.60, 1.55)	0.92 (0.58, 1.47)	0.95 (0.59, 1.52)
Monthly income					
<3,000			Reference	Reference	Reference
3,000–5,000			0.86 (0.51, 1.42)	0.81 (0.49, 1.34)	0.82 (0.49, 1.36)
>5,000			0.62 (0.40, 0.97)	0.58 (0.37, 0.89)	0.60 (0.38, 0.93)
Education					
Illiteracy			Reference	Reference	Reference
Non-illiteracy			0.76 (0.30, 1.90)	0.80 (0.32, 1.99)	0.78 (0.30, 1.97)
Current smoking			0.73 (0.47, 1.12)	0.75 (0.49, 1.15)	0.73 (0.48, 1.13)
Current drinking			0.81 (0.54, 1.22)	0.81 (0.54, 1.21)	0.81 (0.54, 1.21)
Diabetes			1.15 (0.75, 1.77)	1.13 (0.73, 1.74)	1.18 (0.76, 1.82)
Hypertension			1.03 (0.68, 1.56)	1.02 (0.67, 1.53)	1.02 (0.67, 1.55)
Stroke			1.97 (1.27, 3.05)	1.92 (1.24, 2.98)	2.10 (1.35, 3.26)

BMI, body mass index; IPAQ, International Physical Activity Questionnaire; MNA, mini-nutritional assessment. Model 1 and model 2: unadjusted. Model 3, model 4 and model 5: adjusting for age, BMI, IPAQ, MNA score, social contact, monthly income, education level, current smoking, current drinking, diabetes, hypertension, stroke.

**Table 4:** Logistic regression analyses for living alone and osteoporosis with depression in the female.

Characteristic	Model 1	Model 2	Model 3	Model 4	Model 5
	Crude model OR (95% CI)	Crude model OR (95% CI)	Adjusted model OR (95% CI)	Adjusted model OR (95% CI)	Adjusted model OR (95% CI)
Living arrangement					
Living with others	Reference		Reference		
Living alone	2.22 (1.70, 2.89)		1.86 (1.37, 2.54)		
Osteoporosis					
No		Reference		Reference	
Yes		1.52 (1.21, 1.89)		1.21 (0.94, 1.56)	
Living arrangement and osteoporosis					
Living with others and non-osteoporosis					Reference
Living with others and osteoporosis					1.28 (0.96, 1.70)
Living alone and non-osteoporosis					2.11 (1.34, 3.31)
Living alone and osteoporosis					2.21 (1.44, 3.39)
Age			1.02 (0.99, 1.04)	1.02 (0.99, 1.04)	1.01 (0.99, 1.04)
BMI			1.04 (0.99, 1.08)	1.04 (0.99, 1.08)	1.04 (1.01, 1.09)
IPAQ			0.96 (0.93, 0.99)	0.95 (0.93, 0.98)	0.96 (0.93, 0.99)
MNA score			0.79 (0.75, 0.83)	0.80 (0.76, 0.84)	0.79 (0.75, 0.83)
Social contact					
None			Reference	Reference	Reference
Irregular			0.78 (0.58, 1.05)	0.79 (0.59, 1.06)	0.78 (0.58, 1.06)
Regular			0.71 (0.52, 0.96)	0.72 (0.53, 0.97)	0.71 (0.52, 0.97)
Monthly income					
<3,000			Reference	Reference	Reference
3,000–5,000			0.83 (0.61, 1.14)	0.81 (0.60, 1.11)	0.84 (0.61, 1.14)
>5,000			0.80 (0.59, 1.09)	0.67 (0.50, 0.90)	0.82 (0.60, 1.11)
Education					
Illiteracy			Reference	Reference	Reference
Non-illiteracy			0.76 (0.53, 1.08)	0.79 (0.56, 1.12)	0.76 (0.53, 1.08)
Current smoking			1.74 (0.43, 7.11)	1.84 (0.47, 7.31)	1.69 (0.41, 6.89)
Current drinking			0.70 (0.42, 1.19)	0.73 (0.44, 1.23)	0.70 (0.42, 1.19)
Diabetes			0.82 (0.60, 1.12)	0.84 (0.62, 1.15)	0.83 (0.61, 1.14)
Hypertension			0.86 (0.67, 1.11)	0.86 (0.67, 1.12)	0.87 (0.67, 1.13)
Stroke			1.89 (1.42, 2.52)	1.86 (1.39, 2.47)	1.88 (1.41, 2.51)

BMI, body mass index; IPAQ, International Physical Activity Questionnaire; MNA, mini-nutritional assessment. Model 1 and model 2: unadjusted. Model 3, model 4 and model 5: adjusting for age, BMI, IPAQ, MNA score, social contact, monthly income, education level, current smoking, current drinking, diabetes, hypertension, stroke.

## 4 Discussion

To our knowledge, there are few studies that have examined the association between living alone, osteoporosis and the prevalence of depression in a Chinese community-dwelling older population. Overall, females had a higher prevalence of depression than males. The highest depression rate was observed in the living alone and osteoporosis group (Figure 2).

In this study, we found an association with depression only in the combined group of living alone and osteoporosis in males (OR = 3.61, 95%CI = 1.78–7.30). And living alone with or without osteoporosis both was significantly associated with depression in females (OR = 2.11, 95%CI = 1.34–3.31; OR = 2.21, 95%CI = 1.44–3.39, respectively). It’s important to pay attention to the psychological state of people living alone and their bone density, especially the prevalence of depression.

### 4.1 Osteoporosis and depression

Our study found that after adjusting for potential confounders, osteoporosis had no association with depression both in males and females. This result was in accordance with a previous study (21). But some studies revealed contradictory results (15, 23). A cross-sectional study in Iran used the Persian version of the 13-item Beck Depression Inventory (BDI) (23), whereas the USA sample aged ≥50 years used the Patient Health Questionnaire-9 (PHQ-9) (15); both reported an association between osteoporosis and elevated depressive symptoms.

By contrast, our GDS-based research of Chinese community-dwelling older adults (≥60 years) did not show a significant association. The contradiction may result from the difference in the population characteristics; behavioral habits and the different assessment instruments used in the research. People with osteoporosis in our study were less likely to drink, while the Iranian population showed the opposite pattern (23). It is hoped that more future research will investigate the relationship between osteoporosis and depression.

### 4.2 Living alone and depression

Our study revealed a significant association between living alone and depressive symptoms in both sexes, which persisted after adjusting for confounding factors and was consistent with the previous studies (7, 9). The observed association between living alone and depressive symptoms may be partly explained by the absence of social and economic support from family members or friends, a factor previously linked to higher levels of depression (6). Mutual support and a sense of belonging are highly valued, especially in Chinese culture (11, 31). Consequently, Chinese older individuals living alone are more prone to feeling neglected, leading to unhealthy psychological outcomes (31). Moreover, we found that participants living alone tended to have fewer health-promoting behaviors and worse nutritional status (13). One possible explanation was the lack of support and guidance from friends and family, which influence physiology and psychological well-being (9, 32). Surprisingly, in our study, people who lived alone had lower smoking rates, a finding that appeared counter-intuitive but could reflected fewer social cues to smoke. So, further studies considering additional details of living alone need to be conducted in the future.

### 4.3 Living alone, osteoporosis and depression according to sex

Our study suggested that the relationships between living alone, osteoporosis and depressive symptoms were different for females and males. However, what remains consistent is that the combination of living alone and osteoporosis was related to higher levels of depressive symptoms (20).

Surprisingly, osteoporosis was more strongly associated with elevated depressive symptoms in the male subjects living alone (OR = 3.61, *p* < 0.001) than female participants (OR = 2.21, *p* < 0.001). Due to the traditional gender roles, males might have less time to do the housework, and they might be more dependent on females for personal lives and social care, especially in East Asian countries such as China (33, 34). So, even living alone, males are more likely to adopt sedentary lifestyles and participate in less physical activity, especially those with osteoporosis, which increased greatly the ill effects of living alone on depression.

Living alone was associated with various adverse health outcomes—such as decreased physical activity, frailty, falls and higher all-cause mortality—and were also linked to an increased risk of osteoporosis (13, 35). In addition, engaging in outdoor physical activities can help individuals living alone maintain bone mass and reduce the risk of depression (7, 13, 19), partly because physical exercise may increase endocannabinoid concentrations that could ease symptoms of anxiety and depression (36). However, evidence has shown that these individuals are less likely to engage in physical activity and experience a profound decline in activity levels over a two-year follow-up (37). Therefore, maintaining a healthy lifestyle is very important for people living alone to protect bone and mental health.

We also found that living alone was independently associated with more severe depressive symptoms among women. This might be because women are more prone to stress than men (38). Once distressed, females tended to exhibit more anxiety and depression than males when experiencing stress (39). In males, neither living alone nor osteoporosis was significantly associated with depression; rather, the risk elevated only when both conditions were present (OR = 3.61). A possible explanation is the sex differences of stress exposure and stress susceptibility: women frequently develop depression after isolated social stress while men require multiple physical and social stressors (40, 41). On the other hand, older men living alone often enjoy solitude and are more inclined to engage in activities to achieve self-satisfaction and personal growth (42).

Moreover, we observed a pronounced link between socioeconomic inequalities and health. Among men, higher monthly household income attenuated the adverse effects of living alone and osteoporosis on the development of depressive symptoms. Among women, more frequent social participation exerted a stronger protective effect. We speculate that the observed association stems from the fact that adult children are the main source of most Chinese older people from a cultural perspective (31). However, financial independence enables older population to live independently and gain more privacy and autonomy because living alone is not perceived as stressful (9, 10, 39). Active social interactions have shown to improve health promotion and successful aging (43, 44), especially among females, who typically have more extensive social networks than males (31). So, our study suggested that it is necessary to take active aging interventions to help improve the life quality of the older people living alone and ensure their well-being.

### 4.4 Strengths and limitations

The existing research on the separate and combined effects of living alone and osteoporosis on depression is limited. Our study supplemented this information in the Chinese community-dwelling older population. And these findings may provide new insights for clinical care. However, this study had several limitations. Firstly, as this was a cross-sectional study, the causal relationship cannot be determined. Secondly, the participants of this study were almost relatively healthy ones who were able to engage in the annual national physical examination. Thus, our sample may not be comprehensive and representative enough for other regions. Thirdly, we did not consider what effect the transition of living arrangements may have. In the future, further longitudinal follow-up studies are required.

## 5 Conclusion

In conclusion, our study found that living alone and health status have a combined effect in older adults, differing by gender. Our results recommend that more attention should be paid to older individuals living alone. And the sex differences in the association should be considered when formulating and implementing aging policies. Raising monthly income, increasing social participation and their bone density, and other interventions should be implemented to prevent depressive symptoms among them and improve the quality of their lives.

## Data Availability

The datasets generated and analyzed during the current study are not publicly available in order to protect study participant(s) privacy but are available from the corresponding author on reasonable request. Requests to access these datasets should be directed to Qi Guo, guoqijp@gmail.com.

## References

[1] Moussavi S Chatterji S Verdes E Tandon A Patel V Ustun B. Depression, chronic diseases, and decrements in health: results from the world health surveys. Lancet. (2007) 370:851–8. doi: 10.1016/s0140-6736(07)61415-9 17826170

[2] Zhang L Xu Y Nie H Zhang Y Wu Y. The prevalence of depressive symptoms among the older in China: a meta-analysis. Int J Geriatr Psychiatry. (2012) 27:900–6. doi: 10.1002/gps.2821, 22252938

[3] Oh SM Kim HC Ahn SV Rhee Y Suh I. Association between depression and bone mineral density in community-dwelling older men and women in Korea. Maturitas. (2012) 71:142–6. doi: 10.1016/j.maturitas.2011.11.007, 22153349

[4] Xie L-Q Zhang J-P Peng F Jiao N-N. Prevalence and related influencing factors of depressive symptoms for empty-nest elderly living in the rural area of YongZhou, China. Arch Gerontol Geriatr. (2010) 50:24–9. doi: 10.1016/j.archger.2009.01.003 19217674

[5] Van Orden KA Chen S O'Riley A Conwell Y. Course of late-life depression in China is chronic and unremitting. Int J Geriatr Psychiatry. (2014) 30:409–15. doi: 10.1002/gps.4151, 24989837 PMC4282616

[6] Hwang EJ Sim IO. Effect of a comprehensive health care program on blood pressure, blood glucose, body composition, and depression in older adults living alone: a quasi-experimental pretest–posttest study. Int J Environ Res Public Health. (2019) 17:220. doi: 10.3390/ijerph17010220, 31892263 PMC6982085

[7] Xu R Liu Y Mu T Ye Y Xu C. Determining the association between different living arrangements and depressive symptoms among over-65-year-old people: the moderating role of outdoor activities. Front Public Health. (2022) 10:4416. doi: 10.3389/fpubh.2022.954416, 35991056 PMC9386358

[8] Jia Q Duan Y Gong R Jiang M You D Qu Y. Living arrangements and depression of the older adults-evidence from the Chinese longitudinal healthy longevity survey. BMC Public Health. (2023) 23:1870. doi: 10.1186/s12889-023-16730-4, 37759168 PMC10523833

[9] Wu D Liu F Huang S. Assessment of the relationship between living alone and the risk of depression based on longitudinal studies: a systematic review and meta-analysis. Front Psych. (2022) 13:4857. doi: 10.3389/fpsyt.2022.954857, 36111305 PMC9468273

[10] Jeon G-S Choi K Cho S-I. Impact of living alone on depressive symptoms in older Korean widows. Int J Environ Res Public Health. (2017) 14:1191. doi: 10.3390/ijerph14101191, 28991166 PMC5664692

[11] Jiang Y Li M Chung T. Living alone and all-cause mortality in community-dwelling older adults: the moderating role of perceived neighborhood cohesion. Soc Sci Med. (2023) 317:317. doi: 10.1016/j.socscimed.2022.115568, 36442301 PMC9839549

[12] Kojima G Taniguchi Y Kitamura A Fujiwara Y. Is living alone a risk factor of frailty? A systematic review and meta-analysis. Ageing Res Rev. (2020) 59:59. doi: 10.1016/j.arr.2020.101048, 32173535

[13] Lin TY Chen SC Geng JH Tsai HJ. Living alone decreased calcaneus ultrasound T-score in a large Taiwanese population follow-up study. Front Public Health. (2022) 10:1004794. doi: 10.3389/fpubh.2022.1004794, 36276395 PMC9581291

[14] Lee C Park YH Cho B Lee HA. A network-based approach to explore comorbidity patterns among community-dwelling older adults living alone. Geroscience. (2024) 46:2253–64. doi: 10.1007/s11357-023-00987-z, 37924440 PMC10828172

[15] Chen K Wang T Tong X Song Y Hong J Sun Y . Osteoporosis is associated with depression among older adults: a nationwide population-based study in the USA from 2005 to 2020. Public Health. (2024) 226:27–31. doi: 10.1016/j.puhe.2023.10.022, 37988825

[16] Wang H Xia Q Dong Z Guo W Deng W Zhang L . Emotional distress and multimorbidity patterns in Chinese Han patients with osteoporosis: a network analysis. Front Public Health. (2024) 11:2091. doi: 10.3389/fpubh.2023.1242091, 38274525 PMC10808410

[17] Drosselmeyer J Rapp MA Hadji P Kostev K. Depression risk in female patients with osteoporosis in primary care practices in Germany. Osteoporos Int. (2016) 27:2739–44. doi: 10.1007/s00198-016-3584-9, 27026332

[18] Mezuk B Eaton WW Golden SH. Depression and osteoporosis: epidemiology and potential mediating pathways. Osteoporos Int. (2007) 19:1–12. doi: 10.1007/s00198-007-0449-2, 17763997 PMC2776700

[19] Pearce M Garcia L Abbas A Strain T Schuch FB Golubic R . Association between physical activity and risk of depression: a systematic review and meta-analysis. JAMA Psychiatr. (2022) 79:550–9. doi: 10.1001/jamapsychiatry.2022.0609, 35416941 PMC9008579

[20] Shi L Zhou X Gao Y Li X Fang R Deng X. Evaluation of the correlation between depression and physical activity among older persons with osteoporosis: a cross-sectional study. Front Psych. (2023) 14:3072. doi: 10.3389/fpsyt.2023.1193072, 37711420 PMC10499236

[21] Reginster JY Deroisy R Paul I Hansenne M Ansseau M. Depressive vulnerability is not an independent risk factor for osteoporosis in postmenopausal women. Maturitas. (1999) 33:133–7. doi: 10.1016/s0378-5122(99)00057-2 10597877

[22] Stone J Evandrou M Falkingham J. The transition to living alone and psychological distress in later life. Age Ageing. (2013) 42:366–72. doi: 10.1093/ageing/aft006, 23470713 PMC3633366

[23] Kashfi SS Abdollahi G Hassanzadeh J Mokarami H Khani Jeihooni A. The relationship between osteoporosis and depression. Sci Rep. (2022) 12:11177. doi: 10.1038/s41598-022-15248-w, 35778459 PMC9249757

[24] Choi JS Kwak SH Son N-H Oh JW Lee S Lee EH. Sex differences in risk factors for depressive symptoms in patients with COPD: the 2014 and 2016 Korea National Health and nutrition examination survey. BMC Pulm Med. (2021) 21:180. doi: 10.1186/s12890-021-01547-x, 34049523 PMC8161978

[25] Tani Y Kondo N Noma H Miyaguni Y Saito M Kondo K. Eating alone yet living with others is associated with mortality in older men: the JAGES cohort survey. J Gerontol B Psychol Sci Soc Sci. (2017) 73:1330–4. doi: 10.1093/geronb/gbw211 PMC6146753 28093448

[26] Li Y Wang W Zhu L Yang L Wu H Zhang X . Pet ownership, living alone, and cognitive decline among adults 50 years and older. JAMA Netw Open. (2023) 6:9241. doi: 10.1001/jamanetworkopen.2023.49241, 38147332 PMC10751597

[27] Yu X Zheng Y Liu Y Han P Chen X Zhang N . Association of osteoporosis with sarcopenia and its components among community-dwelling older Chinese adults with different obesity levels: a cross-sectional study. Medicine (Baltimore). (2024) 103:e38396. doi: 10.1097/md.0000000000038396, 38875436 PMC11175927

[28] Wu X Hou G Han P Yu X Chen X Song P . Association between physical performance and cognitive function in Chinese community-dwelling older adults: serial mediation of malnutrition and depression. Clin Interv Aging. (2021) 16:1327–35. doi: 10.2147/cia.S315892, 34285477 PMC8285124

[29] Kang L Han P Wang J Ma Y Jia L Fu L . Timed up and go test can predict recurrent falls: a longitudinal study of the community-dwelling elderly in China. Clin Interv Aging. (2017) 12:2009–16. doi: 10.2147/cia.S138287, 29238175 PMC5716394

[30] Wang L Wang X Song P Han P Fu L Chen X . Combined depression and malnutrition as an effective predictor of first fall onset in a Chinese community-dwelling population: a 2-year prospective cohort study. Rejuvenation Res. (2020) 23:498–507. doi: 10.1089/rej.2019.2188, 32303149

[31] Chou KL Ho AHY Chi I. Living alone and depression in Chinese older adults. Aging Ment Health. (2006) 10:583–91. doi: 10.1080/13607860600641150, 17050087

[32] Kim J Choi Y Choi JW Nam JY Park EC. Impact of family characteristics by marital status of cohabitating adult children on depression among Korean older adults. Geriatr Gerontol Int. (2017) 17:2527–36. doi: 10.1111/ggi.13066, 28618150

[33] Honjo K Tani Y Saito M Sasaki Y Kondo K Kawachi I . Living alone or with others and depressive symptoms, and effect modification by residential social cohesion among older adults in Japan: the JAGES longitudinal study. J Epidemiol. (2018) 28:315–22. doi: 10.2188/jea.JE20170065, 29398683 PMC6004365

[34] Baek J Kim G-U Song K Kim H. Decreasing patterns of depression in living alone across middle-aged and older men and women using a longitudinal mixed-effects model. Soc Sci Med. (2023) 317:317. doi: 10.1016/j.socscimed.2022.115513, 36450172

[35] Son H Song S Kim Y Chung J. Patterns of living alone in South Korea compared to other countries: a public health perspective and YouTube topic modeling analysis. Int J Public Health. (2025) 70:1608509. doi: 10.3389/ijph.2025.1608509, 40951592 PMC12427026

[36] Matei D Trofin D Iordan DA Onu I Condurache I Ionite C . The endocannabinoid system and physical exercise. Int J Mol Sci. (2023) 24:1989. doi: 10.3390/ijms24031989, 36768332 PMC9916354

[37] Luo Y Hawkley LC Waite LJ Cacioppo JT. Loneliness, health, and mortality in old age: a national longitudinal study. Soc Sci Med. (2012) 74:907–14. doi: 10.1016/j.socscimed.2011.11.028, 22326307 PMC3303190

[38] Fancourt D Steptoe A Bu F. Trajectories of anxiety and depressive symptoms during enforced isolation due to COVID-19 in England: a longitudinal observational study. Lancet Psychiatry. (2021) 8:141–9. doi: 10.1016/s2215-0366(20)30482-x, 33308420 PMC7820109

[39] Lee CT Chiang YC Huang JY Tantoh DM Nfor ON Lee JF . Incidence of major depressive disorder: variation by age and sex in low-income individuals: a population-based 10-year follow-up study. Medicine (Baltimore). (2016) 95:e3110. doi: 10.1097/md.0000000000003110, 27082549 PMC4839793

[40] Kuehner C. Why is depression more common among women than among men? Lancet Psychiatry. (2017) 4:146–58. doi: 10.1016/s2215-0366(16)30263-2, 27856392

[41] Kendler KS Gardner CO. Sex differences in the pathways to major depression: a study of opposite-sex twin pairs. Am J Psychiatry. (2014) 171:426–35. doi: 10.1176/appi.ajp.2013.13101375, 24525762 PMC3972260

[42] Chen TF Pien LC Fan CS Liang KL Chiu YW. Financial strain and social support as moderators of the relationship between living alone and depressive symptoms in older people. BMC Geriatr. (2024) 24:646. doi: 10.1186/s12877-024-05237-1, 39090539 PMC11293015

[43] Qin Y Liu J Wang R Qi X Jiang S Li J . Can leisure and entertainment lifestyle promote health among older people living alone in China?—a simultaneous equation approach. Front Public Health. (2022) 10:7170. doi: 10.3389/fpubh.2022.967170, 36249231 PMC9558104

[44] Teo AR Choi H Andrea SB Valenstein M Newsom JT Dobscha SK . Does mode of contact with different types of social relationships predict depression in older adults? Evidence from a nationally representative survey. J Am Geriatr Soc. (2015) 63:2014–22. doi: 10.1111/jgs.13667, 26437566 PMC5527991

